# Interdisciplinary Collaboration Between Veterinary and Communication Students to Promote Communication Skills: A Qualitative Pilot Study

**DOI:** 10.3389/fvets.2020.586086

**Published:** 2020-11-24

**Authors:** Asta Tvarijonaviciute, Delfina Roca, Damián Escribano, Lorena Franco-Martínez, Luis J. Bernal, Jose J. Ceron, Silvia Martínez-Subiela, Pedro A. Rojo-Villada

**Affiliations:** ^1^Interdisciplinary Laboratory of Clinical Analysis, Interlab-UMU, Regional Campus of International Excellence ‘Campus Mare Nostrum', University of Murcia, Murcia, Spain; ^2^Department of Information and Documentation, Faculty of Communication and Documentation, University of Murcia, Murcia, Spain

**Keywords:** active learning (AL), collaboration, communication, interdisciplinary learning experiences, veterinary

## Abstract

Interdisciplinary collaborations are increasingly gaining popularity, as are active in higher education and innovative learning strategies. However, relatively little research has been performed related to interdisciplinary learning methodologies in higher education. In the present work, a pilot activity between communication and veterinary students was performed, consisting in performance of mock interviews at a professional television studio. Besides some drawbacks such as low participation rates by veterinary students, the activity was associated with a number of benefits, including enhanced acquirement of communication skills, greater topic-related knowledge assimilation, and reinforced practical application of the theoretical concepts.

## Introduction

Interdisciplinary collaborations are increasingly gaining popularity, as are active learning and innovative forms of learning strategies in higher education (1). Interaction between experts of different areas can help to solve complex problems that would not be possible if only professionals of one domain are involved, leading to an enhanced creativity and improved learning performance (1). Furthermore, different studies emphasize that employers are looking for individuals with a holistic education and who have developed crosscutting skills including the ability to clearly communicate, critical thinking, teamwork, and ability to apply knowledge in multidisciplinary settings (2). Therefore, the ability of knowledge sharing in an interdisciplinary and transdisciplinary way is gaining importance in all areas and should be included in the higher education curricula. This would benefit both the communication skills and the professional possibilities of students (3–5). Nevertheless, relatively little research has been performed related to interdisciplinary learning methodologies in higher education (6).

In particular, students pursuing a communication degree and future journalists must acquire the necessary skills to be able to work as professionals in the field of communication. This implies being able to disseminate topics as specific as those derived from science and research (7). Furthermore, journalists have a direct impact on the opinion of a broad audience and even on decision making, so it is of utmost importance that they know how to accurately and reliably convey news of any origin to the public (8). Therefore, they should have an opportunity to practice the art of interview and preparation of articles about scientific advancement, health, or well-being, among others, during their studies. In this field, they could greatly benefit from interdisciplinary collaboration with veterinary students. Veterinary degree students are taught how to treat and take care of the health and well-being of animals and also trained to take care of public health through food safety and zoonosis control. Therefore, the possibility to practice and interact with veterinary students could provide huge benefits for communication students, as they could get experiences in the implementation of their knowledge and competencies in real situations and thus be better prepared and confident when facing their first work experiences.

Currently, in the majority of science-based degrees such as veterinary medicine, students are taught to share their knowledge, findings, and achievements with other specialists of the same field mainly through presentations in specialized congresses and, although to a lesser extent, through scientific article publishing. In a specific case of veterinary medicine, the curricula frequently include teaching of communication skills but only at the animal owner level. However, they should also be aware of the importance of communicating their knowledge and expertise to a broad audience, the importance of which is becoming increasingly recognized in all fields of research and science (9, 10). In addition, adequate communication of the complex medical topics, research, or other specific knowledge can reach policymakers and specialists from other fields, supporting an interdisciplinary approach for solving real-world problems such as food safety and zoonoses (11).

Furthermore, due to broad competencies, the veterinary profession is considered to be a strong advocate and leader of One Health initiatives, which involve interdisciplinary and transdisciplinary collaboration and knowledge sharing between professionals of different sectors including economists, ecologists, and social scientists; policymakers; and the general public (12). Therefore, it is essential to teach veterinary students how to communicate not just to pet owners or to other specialists and/or scientists from the same field but also to a broad audience and experts from other fields of research (13). Nevertheless, there are still multiple challenges facing scientific dissemination, as only in rare cases is the teaching of these abilities included in the veterinary degree curriculum (14, 15).

In this study, it was hypothesized that a collaborative interdisciplinary activity between communication students and veterinary medicine students could have two-order impacts. The first-order impact could consist of increased assimilation via practical experience of knowledge acquired in the classroom by students of both degrees, while the second-order impact could consist of skill transfer between fields: better public communication skills for veterinary students and better science communication skills for communication students. In addition, the activity could also benefit the educators involved by promoting interaction, collaboration, and exchange of experiences and by developing new teaching skills outside the classroom. Therefore, the main objective of this study was to implement and evaluate a pilot activity between communication and veterinary degree students, consisting of interviews performed at a professional television studio with the aim to investigate the appeal, enjoyment, engagement, and potential benefits of this type of interdisciplinary learning activity for students and educators of the two degrees.

## Materials and Methods

### Participants

In the present pilot study, students of communication and veterinary medicine degrees of the University of Murcia, Spain, academic year 2019/2020 were involved. This project was exempt from human subjects research requirements, as it was conducted as part of a class in a learning institution. Second-year students of journalism degree and 5th-year students of the Joint Program of Journalism and Information and Documentation Degrees (further communication students) attending the compulsory subject “Information Production Systems” were involved in this pilot study. Veterinary medicine degree students who took part were 2nd-year students enrolled in a compulsory course entitled “Nosology and Pathophysiology.” The participation for veterinary students was optional due to the incompatibility of timetables and because the interviews were held in buildings other than the veterinary school buildings. The activity took place in February and March 2020.

### Study Execution

**Figure 1:**
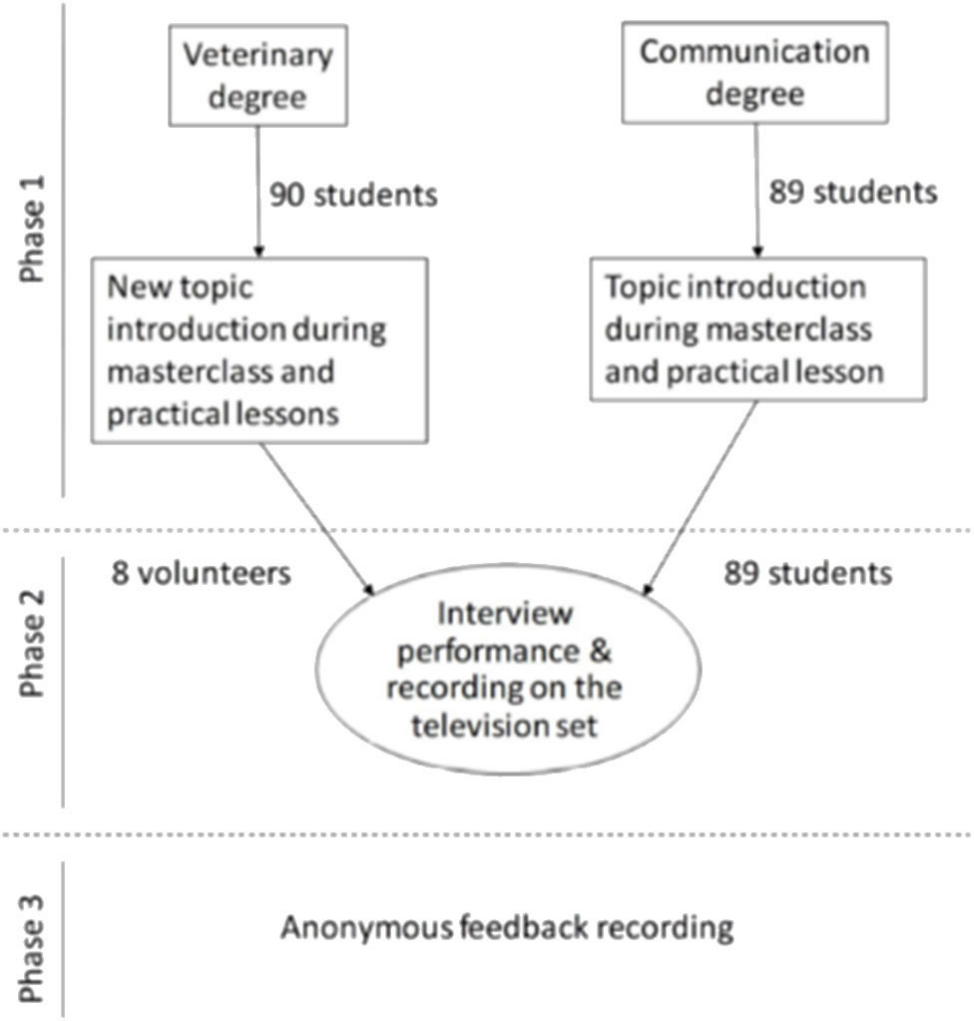
Schematic presentation of the study.

The present study consisted of three phases (Figure 1):

Phase 1—Introduction to the new topic. Veterinary degree students were introduced to the One Health topic in one theoretical class, after which students deepened their topic knowledge with practical lessons and work in groups. Communication students were taught how to plan, prepare, and perform television interviews during the theoretical class and practical lessons. Afterwards, short texts about One Health were shared with communication and documentation students to assist them in preparing questions for the interview.

Phase 2—Interview performance. The interviews were performed as part of a compulsory subject for communication and documentation degree students in “Information Production Systems.” The interviews were performed in the audiovisual resources building AURED of the University of Murcia and were spread over three weekdays. During each day, five (on 2 days) or ten (on 1 day) interviews were performed.

Just before an interview, students of both degrees were introduced and were allowed to discuss the questions prepared by communication degree students. Professors from both degrees were also present in order to support students and, if needed, help with doubts related to questions, responses, and overall execution. Afterwards, in order to compensate for the low number of veterinary medicine students, the interviews were performed as follows: one communication student acted as presenter, and 4–5 students of the same degree acted as collaborators–interviewers, while one to two veterinary student(s) acted as interviewee(s).

Phase 3—Feedback. All students were asked to complete anonymous surveys using an online response system to assess their opinions about the exercise. The survey included four questions related to satisfaction with the implementation of the exercise, expression of the opinion about the increased knowledge acquiring and retention, perception of benefits related to interdisciplinary approach, and their willingness to repeat. To indicate their responses, the students had to score from 1 to 5 (1 meaning total disagreement and 5 total agreement). In addition, a space for free text was left for expression of suggestions for improvement.

In addition, all participating educators from the two degrees were asked to complete surveys consisting of six questions with open responses. The questions were designed to assess the perception of possible benefits, difficulties, and the overall execution of the project.

### Statistical Analysis

Standard statistical programs were used to calculate means and percentages and produce graphs (Excel Microsoft Office 2016, SPSS Statistics 25.0 (IBM Corp., Armonk, NY, USA).

## Results

A total of 97 students participated in the study (Figure 1). As interviews were performed as part of the compulsory course, all communication and documentation students [*n* = 89, with ages between 18 and 25 years; 49.4% (*n* = 44) women] were involved. Meanwhile, eight veterinary students, with ages between 19 and 20 years, 63% (*n* = 5) women, agreed to participate in this pilot study.

**Figure 2:**
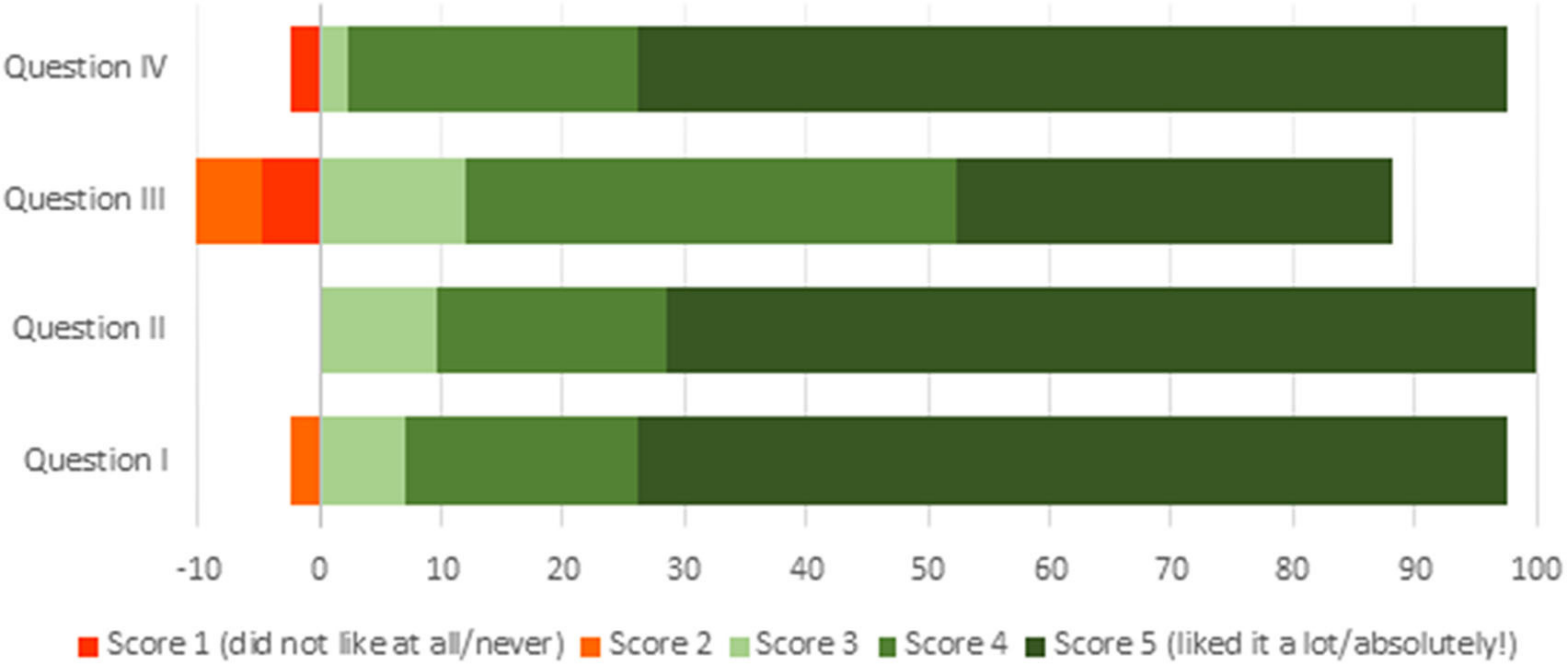
Schematic presentation of the communication and documentation degree students to the anonymous online questionnaire. Questions are indicated with roman numbers. Question I: How much did you like the experience of conducting an interview on a television set? Question II: What is your opinion about participation of students from another faculty in the practice? Question III: Do you think this practice has helped you to strengthen your knowledge of the subject? Question IV: If you had the opportunity, would you repeat the experience?

Percent distribution of the communication students' responses to questions related to the activity is presented in Figure 2. The great majority (71.4%) of communication students indicated that they enjoyed (a score of 5) the experience in the television studio and the collaboration with students from the veterinary faculty and expressed a willingness to repeat the experience if possible. The responses recorded for the question related to classroom-acquired knowledge assimilation varied, 35.7% of students indicated that the activity helped a lot to strengthen their knowledge (score = 5), whereas 40.5% scored 4, 11.9% scored 3, 7.1% scored 2, and 4.8% scored 1 (judging this activity was not useful at all for their knowledge strengthening).

In the same line as communication students, a majority of veterinary students (75%) enjoyed the activity (score = 5), while the remainder scored 4. Eighty-eight percent of veterinary students reported that the activity helped them a lot to deepen their knowledge on the topic (score = 5). Finally, all participating veterinary students (100%) indicated that they would repeat the activity. Furthermore, although only three comments were received, these suggested that students enjoyed the activity and had a good experience (“I really liked everything and I had a great great time”; “We have been treated very well and the experience has been very cool”; “I found everything great and very well organized”).

The educators involved in this project [*n* = 6, 50% (*n* = 3) women] of the two faculties unanimously indicated that the project was very beneficial for both students and educators (Table 1). For communication students, the educators perceived benefits related to putting journalistic specialization into practice; professionally practicing audiovisual genres (reportage and interview) leading to improved expression skills in public; and increasing student self-confidence in front of an audience and specialists of other fields. Similar benefits were reported by educators for veterinary students, including improved communication skills, improved knowledge on the topic, and more awareness of the importance of knowledge assimilation and understanding before information is shared with others. Finally, educators of the two degrees indicated their belief that this kind of activity would be beneficial for students in terms of their future professional task performance, and therefore, it would be desirable to include this interdisciplinary activity in the curriculum of the two specialties. In addition, 100% of educators agreed that this collaborative exercise was highly beneficial for them in terms of exchange of knowledge, ideas and teaching skills, experiences and methodologies within and outside the classroom both virtual and face-to-face. Furthermore, it provided them with a chance to practice and improve competencies that initially are not associated with the profession, such as organization and communication skills, and to identify difficulties of comprehension and expression that students may have in relation to the concepts taught in their subjects.

**Table 1:** Benefits and difficulties identified by the professors participating in the study.

Benefits for students	- new experiences - increased communication capabilities - improved self-confidence against an audience - getting aware of the importance of good knowledge management - topic-related knowledge improvement and understanding - getting experience and skills for activities that adapt to reality - gaining more self-security for their first work experiences - the exchange of information between students of different degrees has benefited the acquisition of new skills - practicing to put into practice audiovisual genres (report, interview, etc.) - getting used to interdisciplinary collaborations and teamwork
Benefits for educators	- new professional experience - improved competencies that are not directly related to the profession, like organization and communication - development of new teaching skills outside the classroom - development of teaching skills both virtual and face-to-face - getting to know the teaching methods in another faculty - exchange experiences with teachers from another faculty - helps to identify difficulties of topic comprehension by students - development of new ideas for future projects
Difficulties	- motivation and organization of veterinary students - coordination of timetables

Identified difficulties were mostly related to the low participation by veterinary degree students, scheduling sessions, and short time available to make the interviews (Table 1).

## Discussion

This pilot educational activity based on voluntary, collaborative, and participatory actions outside the classroom between communication and veterinary degree students was able to fulfill the objectives drawn for the present study based on the perceptions of participants and instructors.

Incorporation of this interdisciplinary learning activity was positively accepted by most participating students. Active learning methodologies are being widely used to substitute for so-called traditional teacher-centered passive learning (16) since active learning methods actively engage students and increase their motivation. These benefits have been attributed to greater assimilation of knowledge and course concepts and the acquirement of enhanced higher-order thinking capabilities (17–19). In agreement with this, the majority of the participating students in both degree programs reported that the activity permitted them to strengthen their knowledge acquired in the classroom. However, given that knowledge was not assessed individually by means of a pre- and post-activity examination (being the main limitations of this activity), this study should be considered a pilot study. And therefore, to objectively demonstrate the benefits of this exercise to students in both programs, future large-population studies should be conducted, in which students in each program would be divided into two groups—one that participates in the activity and one that takes the usual traditional classes (control group), and all groups would be examined pre- and post-activity. This would allow a more precise evaluation of the impact of this educational activity on the assimilation of knowledge by students.

Currently, no scientific discovery is considered as such until it is made known to the scientific community and to society (20). From the informative point of view, it means moving from the deficit model that maintains that knowledge belongs to experts to a more democratic view in which society is taken into account (21). In pursuit of this goal, different countries are adopting policies in order to promote science dissemination and communication to a broad audience. Nevertheless, and as stated above, teaching of scientific communication is rarely included in the curricula of degree programs in the sciences. Therefore, the present study brings new insights and ideas for possible solutions and ways for filling these gaps. In particular, our performed activity permitted students to familiarize and explore in the world of science popularization and dissemination and improve their ability to clearly and effectively convey concepts acquired in the classroom. Veterinary students, with the help of communication students, had to translate the specialized knowledge into a more general language, seeking to accurately inform while capturing the interest and curiosity of the audience, without losing rigor. Given that most veterinary graduates work in private practice, one of the most widely applicable benefits for veterinary students would be enhanced ability to communicate sometimes complex medical topics to clients (pet owners) both inside and outside the clinic. In addition, for those dedicated to research, this course would allow them to acquire skills to communicate their research to nonscientists. Furthermore, both groups of students received immediate feedback on the success of their efforts to explain or express themselves clearly and concisely to nonexperts in their field, favoring interrelationships, both personal and academic.

A few difficulties were faced by educators during the execution of this activity. The majority of these were related to the recruitment and involvement of veterinary students, given that many students could not participate as the schedule of activity and compulsory practices in their program overlapped with that of communication students. Different studies report that veterinary students experience high levels of stress being mainly attributed to the huge workload (22, 23). This, at least in part, could also explain a low volunteer number from the veterinary faculty in the present study. In fact, some of the students that initially showed interest in participation later withdrew, citing the lack of time for studying and preparation for their exams. Another possible reason for their low participation could be a short time for decision making and self-preparation, as the two subjects took place at the beginning of the second semester. Nevertheless, these drawbacks could be amended if timetables of the involved subjects of the two degrees are coordinated and if this activity is included as compulsory in the curricula of the veterinary degree. This suggestion was positively received by the participating educators, who perceived significant benefits to their students from participation in the present exercise.

In conclusion, this interdisciplinary pilot activity showed huge potential for a number of benefits for students and educators of the two degree programs including the amplified acquirement not only of communication skills, but also of a greater topic-related knowledge assimilation among participants and reinforced practical application of the theoretical contents. Nevertheless, future investigations should be performed to accurately and objectively assess outcomes of this exercise on skills of students in both programs.

## Data Availability Statement

The raw data supporting the conclusions of this article will be made available by the authors, without undue reservation.

## Author Contributions

AT, DR, LB, JC, SM-S, and PR-V conceived and designed research. DE and LF-M collected and analyzed data. All authors participated in writing and revising the manuscript. All authors read, correct, and approved the final version of the manuscript.

## Conflict of Interest

The authors declare that the research was conducted in the absence of any commercial or financial relationships that could be construed as a potential conflict of interest.
